# PERCEIVED CONTROL AND CORTISOL STRESS REACTIVITY: VARIATIONS BY AGE, RACE, AND FACETS OF CONTROL

**DOI:** 10.1093/geroni/igz038.3140

**Published:** 2019-11-08

**Authors:** Jin-Hui Wen, Nancy L Sin

**Affiliations:** 1 University of British Columbia, British Columbia, Canada; 2 University of British Columbia, Vancouver, British Columbia, Canada

## Abstract

Greater perceived control is associated with better aging-related health outcomes, and these associations have previously been shown to differ based on sociodemographics. Physiological stress responses—including cortisol reactivity to stressors—may underlie the link between perceived control and health. The goal of this study was to evaluate the associations of perceived control and its facets (personal mastery and perceived constraints) with cortisol reactivity to acute laboratory stressors, in addition to the moderating roles of age and race. Participants (N = 737) ages 25-75 completed a perceived control questionnaire and two lab-based stress tasks. Salivary cortisol was collected pre- and post-stressor exposure. The results showed no main effects of perceived control, personal mastery, nor perceived constraints on salivary cortisol reactivity to stressors. However, age and race moderated the association between perceived constraints and post-stressor cortisol level, adjusting for baseline cortisol, sociodemographics, and health covariates. Among white participants, younger adults who reported higher constraints had elevated cortisol responses compared to those who reported lower constraints, whereas constraints were unrelated to cortisol reactivity among midlife and older adults. Among black participants, perceived control and its subscales were unrelated to cortisol, regardless of age. These findings suggest that older age buffers against the association between constraints and stress reactivity, but this buffering effect is only evident for white participants. Future research on the role of perceived control in stress and health should consider the importance of racial differences, facets of control, and age variations.

